# Mesenchymal Stem Cell Treatment for Steroid Refractory Graft-versus-Host Disease in Children: A Pilot and First Study from Turkey

**DOI:** 10.1155/2016/1641402

**Published:** 2015-12-13

**Authors:** Fatih Erbey, Didem Atay, Arzu Akcay, Ercument Ovali, Gulyuz Ozturk

**Affiliations:** ^1^Department of Pediatric Hematology/Oncology & Bone Marrow Transplantation Unit, Faculty of Medicine, Acıbadem University, Atakent Hospital, 34303 Istanbul, Turkey; ^2^Acıbadem University, Acıbadem Labcell, 34303 Istanbul, Turkey

## Abstract

This study evaluated the efficacy of mesenchymal stem cells (MSCs) from bone marrow of a third-party donor for refractory aGVHD. We report the first experience using MSCs to treat refractory aGVHD in 33 pediatric patients undergoing allogeneic HSCT from Turkey. Totally, 68 doses of bone marrow derived MSCs were infused. The median dose of MSC was 1.18 × 10^6^ cells per kg body weight. Overall, complete response (CR) was documented in 18 patients, partial response (PR) was documented in 7 patients, and no response (NR) was documented in 8 patients. The 2-year estimated probability of overall survival (OS) for patients achieving CR and PR/NR was 63.8% and 29.4%, respectively (*p* = 0.0002). While the cumulative incidence of transplant related mortality (TRM) at day 100 after first MSC infusion was 46.6% in PR/NR patients, there was no any TRM at day 100 after first MSC infusion in CR patients (*p* = 0.001). Twelve patients developed chronic GVHD (cGVHD); eight of them were alive, with five having extensive disease and three having limited disease. In conclusion, MSCs appear to be safe and effective treatment option for pediatric patients with steroid refractory aGVHD. But the efficacy of MSCs on cGVHD in aGVHD patients treated with MSCs seems to be limited.

## 1. Introduction

Graft-versus-host disease (GVHD) is one of the most severe complications in the setting of allogeneic hematopoietic stem cell transplantation (HSCT). Depending on the intensity of the conditioning regimen, the extent of human leukocyte antigen (HLA) match, age of the recipient, and stage of the primary disease, the incidence of GVHD varies from 20% to 70% [1, 2]. Initial treatment with corticosteroids remains the standard for acute GVHD (aGVHD) [3]. However, only 30% to 50% of HSCT recipients experience aGVHD benefit from steroid treatment, in addition recurrence is higher in patients with grades III-IV GVHD [4, 5]. The second-line therapies do not offer significant benefits of aGVHD and have increased risk for infections and toxicities [3]. The 2-year survival is 10% or lower in recipients who experience steroid-resistant GVHD in literature [3–6].

The human mesenchymal stem cells (MSCs) are multipotent progenitor cells that present extensive immunomodulatory properties. The first report of MSCs clinical efficacy for grade IV refractory aGVHD treatment was published in 2004 by Le Blanc et al. [7]. Later, a multicenter study demonstrated the efficacy of directed donor MSCs in the treatment of adult and pediatric patients with steroid refractory aGVHD [8]. The complete MSC response varies from 15% to 55% in treated aGVHD patients [9–11]. The incidence and severity of cGVHD are rarely reported in refractory aGVHD patients treated with MSCs. Here, we describe the first experience of MSC use for the treatment of pediatric patients with steroid refractory aGVHD from Turkey.

## 2. Materials and Methods

### 2.1. Study Design

The study was a retrospective, single center study and it was approved by the local ethical committee. Data were collected from patients' files and written informed consent according to the declaration of Helsinki was obtained in all cases from parent.

### 2.2. Patients

Thirty-three patients were enrolled between November 2011 and June 2015. The patients were eligible if they had developed grades III-IV aGVHD as defined and graded by international criteria [12], which were treated with standard first-line treatment with corticosteroids and thereafter at least one second-line therapy. Steroid resistant aGVHD was defined as either no response to steroid treatment (minimum 2 mg/kg/d methyl-prednisolone or equivalent) lasting at least 7 days or progression during treatment of at least one grade within the first 72 hours. Prophylactic therapy with either cyclosporine (CsA) or tacrolimus and/or mycophenolate mofetil (MMF) was continued at therapeutic dose level. MMF could not be used in patients with gastrointestinal system GVHD because of their intestinal toxicity and suspicion of bioavailability. Safety endpoints included infusional toxicity, adverse reactions, formation of ectopic tissue, infection, and death. Patients with multiple MSC infusions were received MSC at two-week interval; they were evaluated for response at 28 days after MSC infusion. Complete response (CR) was defined as disappearance of all symptoms due to aGVHD, while partial response (PR) was defined as an improvement of at least one overall grade. No response (NR) was defined as no change in aGVHD grade and/or progressive worsening of aGVHD. Overall survival (OS) was defined as the probability of survival, regardless of disease status; surviving patients were censored at time of last follow-up, while only death was considered an event. Transplant related mortality (TRM) was defined as the probability of dying without previous occurrence of relapse, which was considered to be the competing event.

### 2.3. MSC Preparation for Clinical Use

#### 2.3.1. Cell Source

Cell source is third-party bone marrow of the volunteer who has informed consent form about bone marrow donation.

#### 2.3.2. Production Method

The mononuclear cells were isolated from 20 mL bone marrow with density gradient centrifugation method. Cells were suspended and plated into culture flasks in DMEM-LG containing 10% human serum and 1% penicillin (complete media) and were cultured at 37°C in 5% CO_2_. Culture medium was changed with fresh medium once every 3 days and waited for 70% confluency, nearly 14 days. At the end of primary culture, medium was removed from flasks. Trypsin/EDTA 0.25% solution was added to the flasks and incubated in incubator for 5 minutes. After neutralization, the trypsin reaction with 1 mL human serum, cells were collected in a tube and centrifuged with PBS at 400 g. This washed step was repeated twice. Cells were resuspended in fresh complete medium and seeded in larger amount of flasks, after first^−^passage cells were trypsinized, washed, and resuspended in complete media and frozen with cryoprotectant, containing 7.5% DMSO, 3% HES, and 1% human albumin. Upon request, the frozen cells were thawed and cultured in the same condition. After the cells reach 70% confluence, medium was removed from flasks. Cells were trypsinized, washed, and resuspended in Ringer's lactate. 2 × 10^6^ cells/mL in Ringer's lactate containing 1% human serum albumin were transferred in vials with the temperature controlled bag in 12 hours. The product was used in 24 hours. Release criteria included lack of detectable microbial contamination (aerobic or anaerobic bacteria, fungi, and mycoplasma) according to European pharmacopoeia, cell viability ≥ 90%, endotoxin levels in the final product ≤ 5 EU/kg, and cell characterization with positive expression of CD73, CD90, and CD105 and with negative expression of CD14, CD34, CD45, and HLA-DR [13, 14].

### 2.4. Statistical Analysis

SPSS (Statistical Package for Social Sciences), version 13, statistical package program was used for the analysis of the data. Chi square test was used for the comparison of the discrete variables. *p* < 0.05 was regarded as statistically significant. Survival rates were compared using Kaplan-Meier method. The comparisons were performed using log-rank test. *p* < 0.05 was regarded as statistically significant.

## 3. Results

The median age of the patients was 7 years (range: 3–18 years) with 17 males and 16 females. The patient characteristics, conditioning regimens, and GVHD prophylaxis are listed in Table 1. The organ involvement and grade of aGVHD are shown in Table 2.

Totally, 68 doses of bone marrow derived MSCs were infused. MSCs were obtained from HLA-identical family donor (*n* = 1) and third-party HLA-mismatched donors (*n* = 67). The median duration between the diagnosis of aGVHD and initiation of MSCs therapy was 18 days (range: 5–88). Nine patients received one dose, fifteen received two doses, seven received three doses, and two received four doses. The median dose of MSC was 1.18 × 10^6^ (min-max range 0.54–2.80 × 10^6^) cells per kg body weight. No patients had side-effects during or immediately after infusions of MSCs.

Overall, CR was documented in 18 patients, PR was documented in 7 patients, and NR was documented in 8 patients. One patient, presented with grade IV aGVHD and invasive pulmonary fungal infection, died 9 days after MSC treatment and he was classified as a nonresponder. 32 patients had skin involvement: CR in 24 (75%), PR in 7 (21.8%), and NR in 1 (3.2%) patients. 29 patients had gastrointestinal involvement: CR in 19 (65.5%), PR in 3 (10.3%), and NR in 7 (24.2%) patients. 14 patients had liver involvement: CR in 5 (35.7%), PR in 2 (14.3%), and NR in 7 (50%) patients. Overall and organ specific responses rates are shown in Table 3.

The median duration between the diagnosis of aGVHD and initiation of MSCs therapy was 18 days (range: 5–88). We composed two groups on the basis of the median time. According to this setting, we defined early (between 5 and 21 days after the beginning of aGVHD treatment) and late (between 22 and 88 days after the beginning of aGVHD treatment) group. The early and late group consisted of 20 and 13 patients, respectively. There was no difference in the two groups in terms of CR and OS.

**Figure 1:**
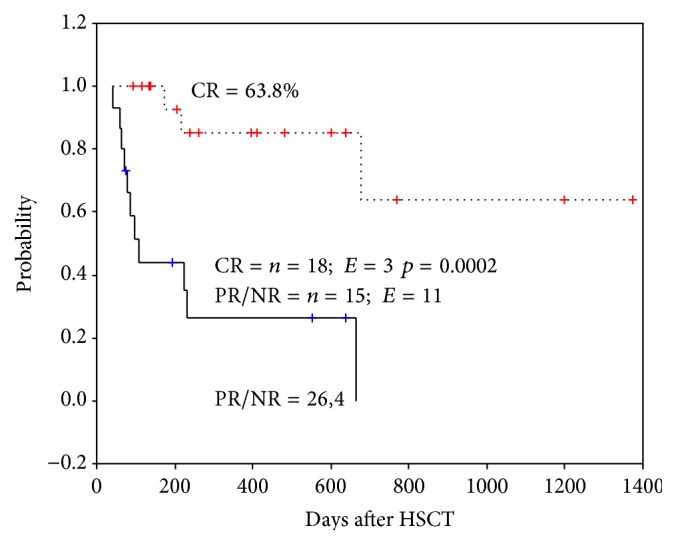
Two-year Kaplan-Meier estimate of overall survival according to response to mesenchymal stromal cell treatment. CR: complete response, PR/NR: partial or nonresponse, *n*: number, and *E*: event.

Nineteen of 33 patients (57.6%) were still alive with a median follow-up of 335 days (range: 41–1319) after first MSCs infusion. The 2-year estimated probability of OS for patients achieving CR and PR/NR was 63.8% and 26.4%, respectively. The difference between two groups was found statistically significant (*p* = 0.0002) (Figure 1). While the cumulative incidence of TRM at day 100 post first MSC infusion was 46.6% in PR/NR patients, there was no any TRM at day 100 post first MSC infusion in CR patients. The difference between the two groups was found statistically significant (*p* = 0.001). The cumulative incidence of TRM at 2 years after MSC infusion was 11.7% for patients with CR compared to 73.3% for patients with PR/NR (*p* = 0.001).

Twelve patients developed cGVHD, of whom eight are alive. Of these 8 surviving children, 5 have extensive disease and three have limited disease, and their immunosuppressive drug treatments are ongoing. Fourteen of 33 patients were dead, and the median time to death was 73 days (range: 9–644) from the initial MSC therapy. The causes of death are listed in Table 2.

## 4. Discussion

MSCs, also known as mesenchymal stromal/stem cells, are nonhematopoietic and a form of multipotent adult stem cells that can be isolated from many tissues, such as bone marrow (BM), adipose tissue, and umbilical cord. They were originally defined as self-renewing, multipotent progenitor cells with multilineage potential to differentiate into other types of cells of mesoderm origin as well as cells of nonmesodermal origin. MSCs provide not only stromal support for hematopoietic stem cells in the BM but also have potent immunosuppressive and anti-inflammatory effects, which are significant clinical implications in HSCT. MSCs suppress T-cell proliferation and increase the number of regulatory T cells. In addition, MSCs inhibit function of B cells, natural killer cells, and dendritic cells. The immunosuppressive capacity of MSCs is enhanced under inflammatory conditions in the presence of the proinflammatory cytokines interferon- (IFN-) *γ*, tumor necrosis factor-*α*, and interleukin- (IL-) 6. MSCs target neutrophils and monocytes under noninflammatory conditions, but they attract monocytes, dendritic cells, T cells, and natural killer cells under inflammatory conditions [15].

GVHD is a severe inflammatory condition, which results from immune-mediated attack of recipient tissue by donor T cells contained in the allogeneic graft. In contrast to aGVHD, the pathophysiology of cGVHD is poorly understood. While aGVHD is characterized by direct cytotoxic effects of donor T cells on recipient tissues, activation of antigen-presenting cells, and an inflammatory cascade that produces cytokines, including IL-1, IL-6, IL-12, IFN-*γ*, and tumor necrosis factor-*α*, cGVHD is characterized by autoimmune-like dysregulation. Although donor T cells clearly play a critical role in the initiation and maintenance of alloimmunity, many laboratory and clinical studies have shown that donor B cells also play an important role in the pathophysiology of cGVHD [16–18].

In this study, we report the first experience using MSCs to treat refractory aGVHD in children undergoing allogeneic HSCT from Turkey. We analyzed 33 pediatric cases. Similarly, in the previously published studies reporting on pediatric patients by Le Blanc et al., Lucchini et al., and Prasad et al., the number of children analyzed was 17, 12, and 11, respectively [6–9]. So far, Ball et al. reported largest pediatric cohort with 37 cases [19]. The MSC treatment caused a CR in 54.5% of our patients and a PR in 21.2% of cases. Similar results were reported by Le Blanc et al. [8] (55% CR, 16% PR), Lucchini et al. [9] (23.8% CR, 47.6% PR), Prasad et al. [6] (58% CR, 17% PR), and Ball et al. [19] (65% CR, 21.6% PR). Our patients received MSCs at two-week interval. We found similar efficacy to those receiving twice a week MSC infusions over 4 weeks [6, 8, 20]. We have achieved 75%, 65.5%, and 35.7% CR in skin, gastrointestinal, and liver involvement, respectively. Ball et al. [19] reported 57%, 52.6%, and 44% CR in skin, gastrointestinal, and liver involvement, respectively. Prasad et al. [6] reported 100%, 75%, and 25% CR in skin, gastrointestinal, and liver involvement, respectively. Although we have found similar efficacy, we suggest that, in patients with PR or NR, it could be better to continue MSC infusions over 4 week.

The patients achieving CR had a much better OS than those who achieved a PR/NR in this study. Similar to our findings, Prasad et al. reported that 2-year estimated probability of OS for patients achieving CR and non-CR was 68% and 0%, respectively [6]. Ball et al. reported that a 6-year estimated probability of OS for patients achieving CR and PR/NR was 65% and 0%, respectively [19]. They found that the cumulative incidence of TRM at day 100 after first MSC treatment was 9% in patients who achieved CR as compared to 47% in those with either PR or NR. In addition, the cumulative incidence of TRM at 6 years after MSC treatment was 17% for patients with CR compared to 69% for patients with PR/NR. In current study, the cumulative incidence of TRM at day 100 after first MSC infusion was 46.6% in PR/NR patients, there was no any TRM at day 100 after first MSC infusion in CR patients, and the cumulative incidence of TRM at 2 years after MSC infusion was 11.7% for patients with CR compared to 73.3% for patients with PR/NR.

The optimal timing for administration of MSC to best ameliorate the symptoms of GVHD has been investigated by Polchert et al. in a murine GVHD model [21]. MSCs were introduced into this model concurrently with bone marrow infusion, or 2, 20, or 30 days after one marrow infusion. Mice died of the symptoms of aGVHD when they received no MSC or at early time point with bone marrow infusion, or 30 days after bone marrow infusion (late time point). However, when MSCs were administered 2 or 20 days after bone marrow infusion, significantly increased survival rates were observed indicating that the administered MSC acted to ameliorate the symptoms of the aGVHD. Similar observations were reported in other studies. Prasad et al. [6] reported that the median duration between the diagnosis of aGVHD and the therapy with MSC was 46 days (range: 18–181). They stated that a long gap of 6 to 7 weeks between the diagnosis of aGVHD and the therapy with MSC may be responsible for some of the therapeutic failures and poor outcome in those not achieving CR. On the other hand, Ball et al. [19] showed that children treated early (between 5 and 12 days after the beginning of aGVHD treatment) with MSC more frequently obtained CR than those treated late (between 13 and 85 days after the beginning of aGVHD treatment), and there was a lower 6-year TRM in children treated early compared to those receiving MSC late. Finally, they suggested that early treatment with MSC may be associated with reduced TRM and better OS. In this study, the median duration between the diagnosis of aGVHD and the initiation of MSC therapy was 18 days (range: 5–88) and we composed early and late group as mentioned above. The number of patients in our cohort was relatively small, so we did not found any difference in the two groups in terms of CR and OS. Like Ball et al. [19], we also think that early treatment with MSC is more beneficial in the management of aGVHD.

The role of MSC treatment in aGVHD has been an increasing interest in allogeneic HSCT. The clinical responses of MSCs to treat cGVHD are controversial. The majority of patients showed only partial or mixed responses, suggesting that MSC may not be a potent immunomodulator in a cGVHD environment [22–24]. In our study, twelve patients (12/33) developed cGVHD, eight of them were in CR group and four were PR/NR group. Zhao et al. found that the incidence and severity of cGVHD in aGVHD patients undergoing MSC treatment were lower than those without MSC treatment [25]. Peng et al. found in their studies that CD19^+^ B cells decreased, but the frequencies of CD5^+^ regulatory B cells, CD27^+^ memory B cells, and pregerminal center B cells increased in CR and PR provided in cGVHD patients after MSC treatment [26, 27]. They stated that it is worth further study to know whether MSCs also ameliorated cGVHD by modulating B cells.

In conclusion, MSCs therapy appears to be safe and effective treatment option for pediatric patients with steroid refractory aGVHD, but the role of MSCs to treat cGVHD is still controversial. To date, a variety of dosing schedules has been used; however, the optimal treatment method should be determined. Based on the results of clinical studies, to improve the safety and efficiency of MSC therapy, studies of specific markers that identify MSCs, cell dose, and the timing are crucial and must continue. Therefore, more definitive studies and longer follow-ups during clinical trials are necessary to assess the long-term efficacy and toxicity associated with MSC use.

## Figures and Tables

**Table 1:** Patients and HSCT characteristics.

Age (years)	Median: 7 Range: 3–18

Sex female/male	16/17

Diagnosis	*N*
Malign diseases	16
Nonmalign diseases	17

Type of transplantation	
MUD	18
MSD	7
MRD	4
Haploidentical	4

Source of stem cells	
BM	29
PBSC	4

Conditioning regimen	
Myeloablative	24
Nonmyeloablative	9

GVHD prophylaxis	
CsA	13
CsA + Mtx	11
CsA + MMF	6
MMF	3

MSD: match sibling donor; MRD: match related donor; MUD: match unrelated donor; BM: bone marrow; PBSC: peripheral blood stem cell; CsA: cyclosporine A; Mtx: methotrexate; MMF: mycophenolate mofetil.

**Table 2:** Characteristics of GVHD and response to MSC therapy.

Patients	Skin/GI/liver (stages)	aGVHD (grade)	Response	Follow-up	Cause of death
1	2/4/0	IV	CR	Alive with extensive chronic GVHD	
2	2/2/2	III	CR	Death	Bacterial infection
3	2/4/0	IV	NR	Death	Extensive chronic GVHD
4	4/3/0	IV	CR	Alive, no GVHD	
5	3/3/0	III	CR	Death	Extensive chronic GVHD + infection
6	4/4/0	IV	CR	Death	Leukemia relapse
7	2/4/4	IV	NR	Death	aGVHD + infection
8	4/2/2	IV	NR	Death	aGVHD + infection
9	3/4/3	IV	CR	Alive, no GVHD	
10	4/4/3	IV	CR	Alive, no GVHD	
11	4/4/3	IV	PR	Death	aGVHD + infection + VOD
12	4/4/1	IV	CR	Alive with limited chronic GVHD	
13	3/4/2	IV	PR	Alive, no GVHD	
14	4/0/0	IV	PR	Alive with limited chronic GVHD	
15	4/4/0	IV	CR	Alive with limited chronic GVHD	
16	0/4/0	IV	CR	Alive, no GVHD	
17	4/2/4	IV	PR	Death	aGVHD + infection
18	4/4/4	IV	NR	Death	aGVHD
19	4/0/0	III	CR	Alive with extensive chronic GVHD	
20	4/4/3	IV	NR	Death	aGVHD
21	4/4/0	IV	NR	Death	aGVHD + infection
22	4/4/0	IV	CR	Alive with extensive chronic GVHD	
23	4/4/0	IV	CR	Alive, no GVHD	
24	4/4/3	IV	PR	Alive, no GVHD	
25	3/3/4	IV	NR	Death	aGVHD + infection + VOD
26	3/4/0	IV	PR	Death	aGVHD + infection
27	2/4/0	IV	CR	Alive with extensive chronic GVHD	
28	4/0/0	III	CR	Alive, no GVHD	
29	2/4/0	IV	CR	Alive, no GVHD	
30	4/0/0	III	CR	Alive, no GVHD	
31	3/4/4	IV	NR	Death	aGVHD
32	4/4/0	IV	PR	Alive, with aGVHD	
33	2/4/0	IV	CR	Alive, no GVHD	

GVHD = graft-versus-host disease; GI = gastrointestinal; CR = complete response; PR = partial response; NR = no response; VOD = venoocclusive disease.

**Table 3:** Overall and organ specific response rate.

	*n* (%)
Overall response rate	
CR	18/33 (54,5)
PR	7/33 (21,2)
NR	8/33 (24,3)

Response rate according to organ involvement	

Skin (32 patients)	
CR	24/32 (75,0)
PR	7/32 (21,8)
NR	1/32 (3,2)

GI (29 patients)	
CR	19/29 (65,5)
PR	3/29 (10,3)
NR	7/29 (24,2)

Liver (14 patients)	
CR	5/14 (35,7)
PR	2/14 (14,3)
NR	7/14 (50,0)

GI: gastrointestinal; CR: complete response; PR: partial response; NR: no response.
